# A Target-Specific, “Eco-Friendly” Experimental Setup for Small Field Proton Irradiation

**DOI:** 10.1016/j.ijpt.2026.101953

**Published:** 2026-08-11

**Authors:** Isabella Colizzi, Ylva Bornhauser, Antony J. Lomax, David Meer, Serena Psoroulas

**Affiliations:** 1 Center for Proton Therapy, Paul Scherrer Institute, Villigen PSI, 5232 Switzerland; 2 Department of Physics, ETH Zurich, Zurich, 8092 Switzerland; 3 Department of Radiation Oncology, University Hospital Zurich, (USZ), University of Zurich (UZH), Raemistrasse 100, Zurich, CH-8091 Switzerland

**Keywords:** Passive scattering, Patient-specific, 3D printing, FLASH

## Abstract

**Purpose:**

Increasing interest in proton FLASH radiotherapy has led to a focus on target-specific (TS) devices and passive scattering (PS) techniques. However, these techniques often require expensive, time-consuming custom devices like single-use collimators and compensators. Our study aims to improve this by creating a flexible TS-PS setup adaptable to various needs, specifically for small target structures.

**Methods:**

The collimating and compensating elements are made from 3D-printed polylactide containers filled with reusable copper or polypropylene spheres. We designed these TSPS elements using the modified Python package Porespy and conducted simulations in Tool for Particle Simulation Monte Carlo. We evaluated the collimator’s performance by comparing a solid collimator with our proposed approach. As a feasibility study, we developed 2 ”eco-friendly” PS setups for irradiating a simple spherical target and a complex small structure, a murine brain tumor.

**Results:**

We validated the computational model and MC simulations through depth dose curve and beam size measurements, demonstrating an agreement within a few percent. The “eco-friendly” collimators effectively collimated the beam, but we observed a dose halo at high energies due to protons not being stopped by the plastic bore; using higher-density materials can address this issue. We designed and validated the 2 “eco-friendly” setups via dose measurements and demonstrated that we could effectively compensate and collimate the beam to conform the dose to the target shape. The total time required to print the setup was under 45 minutes, and the filament cost was under $1.

**Conclusions:**

This innovative, “eco-friendly” approach using 3D printing allows for quick production of TS shapes, reducing waste and costs while enhancing conformity and improving the efficiency of PS treatments.

## Introduction

The emerging interest in preclinical proton irradiation, particularly for small, target-specific (TS) experiments, including but not limited to FLASH radiotherapy,^1^, ^2^ has increased the need for flexible and efficient TS devices utilized in passive scattering (PS), including compensators, collimators, and energy modulators. Furthermore, radiobiological advancements in the field necessitate extensive preclinical experiments, typically targeting small regions and implanted tumors in murine models. Producing TS-PS devices is resource-intensive, and they often become non-reusable after initial use. Researchers often repurpose older devices to reduce costs and preparation time, which can lead to suboptimal solutions.

In recent years, 3D printing has become an important tool in medical physics, offering innovative solutions for quality assurance phantoms, bolus, immobilizers, and field-shaping and patient-specific devices.^3^, ^4^, ^5^ 3D printing offers advantages such as customization, reduced costs, and the ability to be manufactured in-house when an institute has access to a 3D printer. One downside is that printing materials are usually based on plastic because metal can be costly and requires specialized equipment, so it is seldom used to replace metal components like collimators. Additionally, current designs are limited to patient-specific, single-use applications. To address this challenge, a solution proposed for electron radiotherapy^6^ involves 3D-printed cutouts made of plastic shells filled with 2 mm tungsten ball bearings. A similar idea using alloy granules to fill up a compensator hollow was proposed for intensity-modulated radiotherapy.^7^ These solutions enable the creation of fast, reusable, TS devices with the only disposable component being the 3D-printed container, making them therefore low‑waste and reusable in an “eco‑friendly” sense.

Our study investigates, for the first time, the feasibility of “eco‑friendly” hollow 3D-printed compensators and collimators filled with plastic or copper spheres for PS proton therapy irradiation of small targets. Understanding the impact of such porous-like devices on dose distribution and developing a Monte Carlo simulation to accurately predict outcomes is crucial for optimizing the experimental setup. This innovative solution has the potential to reduce waste, lower costs, improve dose conformity, enhance the efficiency of PS experiments, and reduce their environmental impact.

## Methods

### Design and 3D printing of “eco-friendly” target-specific passive scattering devices

The PS devices were designed to reduce the need for TS components by dividing them into 2 distinct parts. The reusable component consists of small metal and plastic spheres. In contrast, the TS hollow components were manufactured using standard 3D printing technologies, which helps to maintain relatively low costs. These hollow containers, either for collimators or compensators, were printed with polylactide (PLA, 25$/kg) filament with commercially available 3D printers, “Original Prusa i3 MK3S+” and “MK4S.”^8^ In addition, we performed one measurement printing the collimator bore with Copper-filled Metal Composite HTPLA filament,^9^ a PLA filament enriched with copper powder, commercially available and compatible with conventional filament printers, to test the impact of the material on the bore design.

The hollow container designs were created in Python for simple shapes and using 3D Slicer for the more complex case of a murine tumor. The designs were exported as Stereolithography binary format (STL) files and imported into Prusa Slicer.

The hollow containers were manually filled with polypropylene (PP) spheres^10^ to serve as compensator devices and with copper (Cu) spheres as collimating devices (PP: 1.191 mm radius, 0.007 g weight; Cu: 1.000 mm radius, 0.038 g weight). The spheres’ radii were selected to be small enough not to affect the dose distribution but not so small as to make handling difficult.

### Experimental setup

All measurements were performed in PSI Gantry 2. In Table 1, we summarize the performed measurements and the different setups employed.

**Table 1:** Summary of the measurements.

Measurement goal	Setup	Instrumentation	Measured quantity
Simulation versus measurement	(1) PLA hollow boxes with PP and Cu spheres. Single spot delivery with multiple energies (100-220 MeV).	QA phantom	DDC and trans-verse dose profile
Collimator performance	(2) PLA (and HTPLA) hollow collimators with Cu spheres, and a solid Cu collimator.Scattered 90-120 MeV beam.	CCD Camera	Transverse dose profile
Feasibility of PS delivery 1	(3) PLA hollow collimator with Cu spheres and compensator with PP spheres designed for a spherical target. Scattered 120 MeV beam.	CCD Camera and PMMA slabs	Transverse dose profile
Feasibility of PS delivery 2	(3) PLA hollow collimator with Cu spheres and compensator with PP spheres designed for a small tumor target. Scattered 110 MeV beam.	Gafchromic films in PMMA slabs	Longitudinal and transverse dose profile

To test the agreement between our simulation workflow and measurements, we performed beam size and depth dose curve (DDC) measurements using the PSI daily QA phantom. The phantom is equipped with 2 scoring planes to measure the dose profile perpendicular to the beam direction and a multilayer ionization chamber for the integral DDC. The multilayer ionization chamber consists of 128 plates with a water equivalent thickness of 2.3640 mm each. Gafchromic films interleaved in PMMA slabs. In Setup 2 and 3, we utilized a charge-coupled device camera attached to a scintillating screen to measure the beam profile after collimation (0.4 mm resolution). In Setup 3, we also measured the dose distributions using Gafchromic films interleaved in PMMA slabs.

### Simulation workflow

All MC simulations in this work were performed using TOPAS (Tool for Particle Simulation,^11^, ^12^ Version 3.9), previously validated for PSI Gantry 2.^13^ We utilized the suggested default modular physics list^11^ (G4EMLOW6.48, G4NDL4.5, PhotonEvaporation3.2, RadioactiveDecay4.4, G4SAIDDATA1.1, G4NEUTRONXS1.4, G4PII1.3, G4ABLA3.0, G4ENSDFSTATE1.2.1, G4TENDL1.0) and the simulations were performed with 10^8^ particles. The scorer had a resolution of 0.1 mm in the lateral direction.

**Figure 1:**
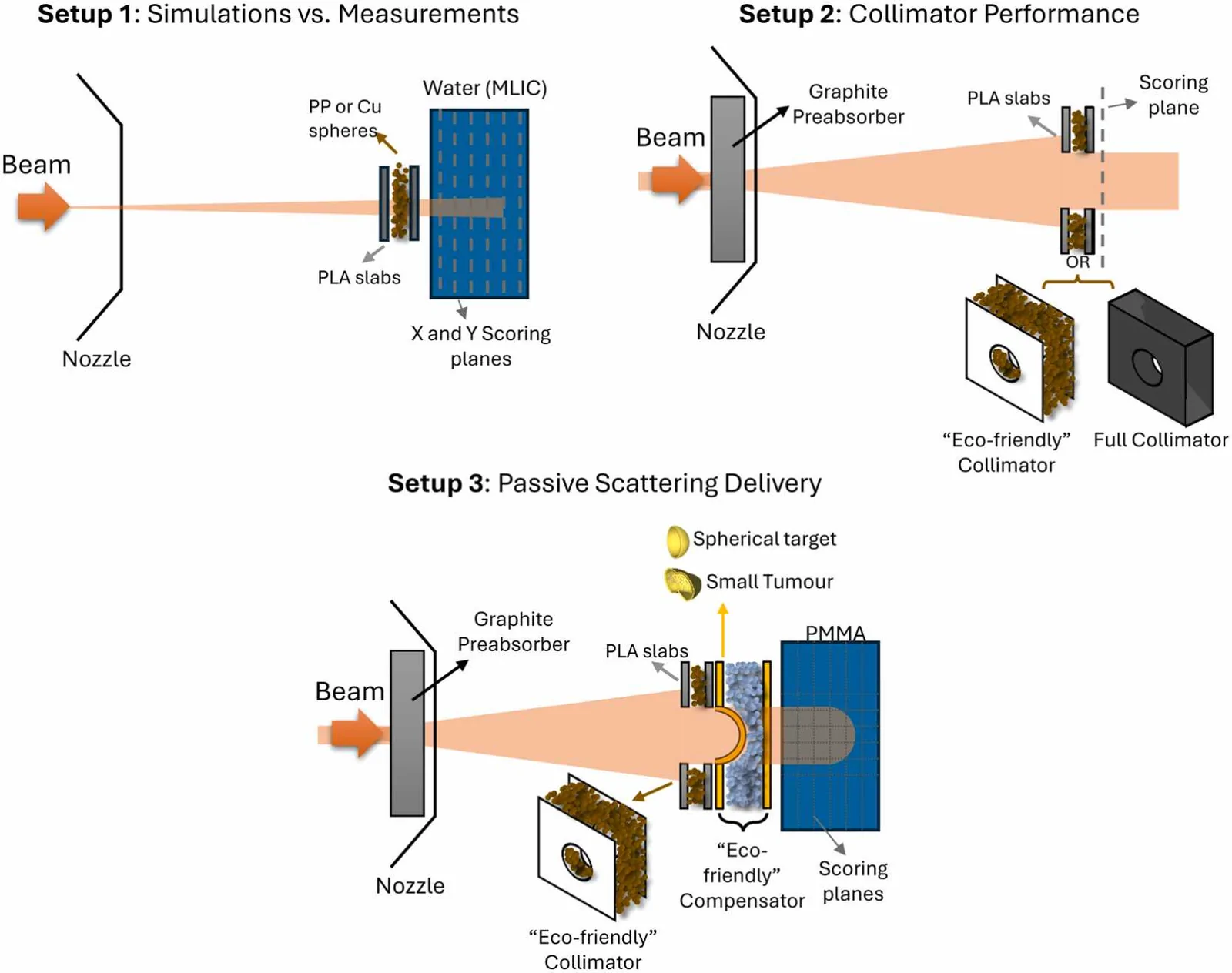
TOPAS setup for the 4 measurements summarized in Table 1.

The hollow containers were represented as simple PLA slabs or, for complex geometries such as the surface of the compensator for mouse irradiation, imported as STL files, as depicted in Figure 1 (Setup 3). The mouse CT was performed with a high-resolution small-animal CT camera used in a previous study,^14^ and the tumor contour was arbitrarily drawn in 3D Slicer.^15^ The target was converted into an STL file and exported. The compensator shape was defined by subtracting a simple STL box with the STL tumor volume using a Boolean difference operation, and then hollowing the resulting structure by removing its internal volume, leaving only the walls for printing the hollow container. To simulate the infill, we used and modified the function “pseudo gravity packing” from Porespy, Quantitative Image Analysis of Porous Materials.^16^ The original algorithm fills a 3D box with spheres by dropping each new sphere to the lowest point possible, simulating the effect of gravity. We modified this function to facilitate the filling of more complex shapes using the Open3D Python package^17^ and to enable the export of an appropriate file for simulations. TOPAS supports the inclusion of STL files, but even when undersampled, STL files representing thousands of spheres can be quite large. This can complicate the loading of geometry and the simulation of particle interactions, often resulting in lengthy loading times and extended execution durations for the simulations. Therefore, we have chosen to export the spheres directly as a list of TOPAS geometry components, specifically TsSphere.

## Results

### Simulations versus measurements

**Figure 2:**
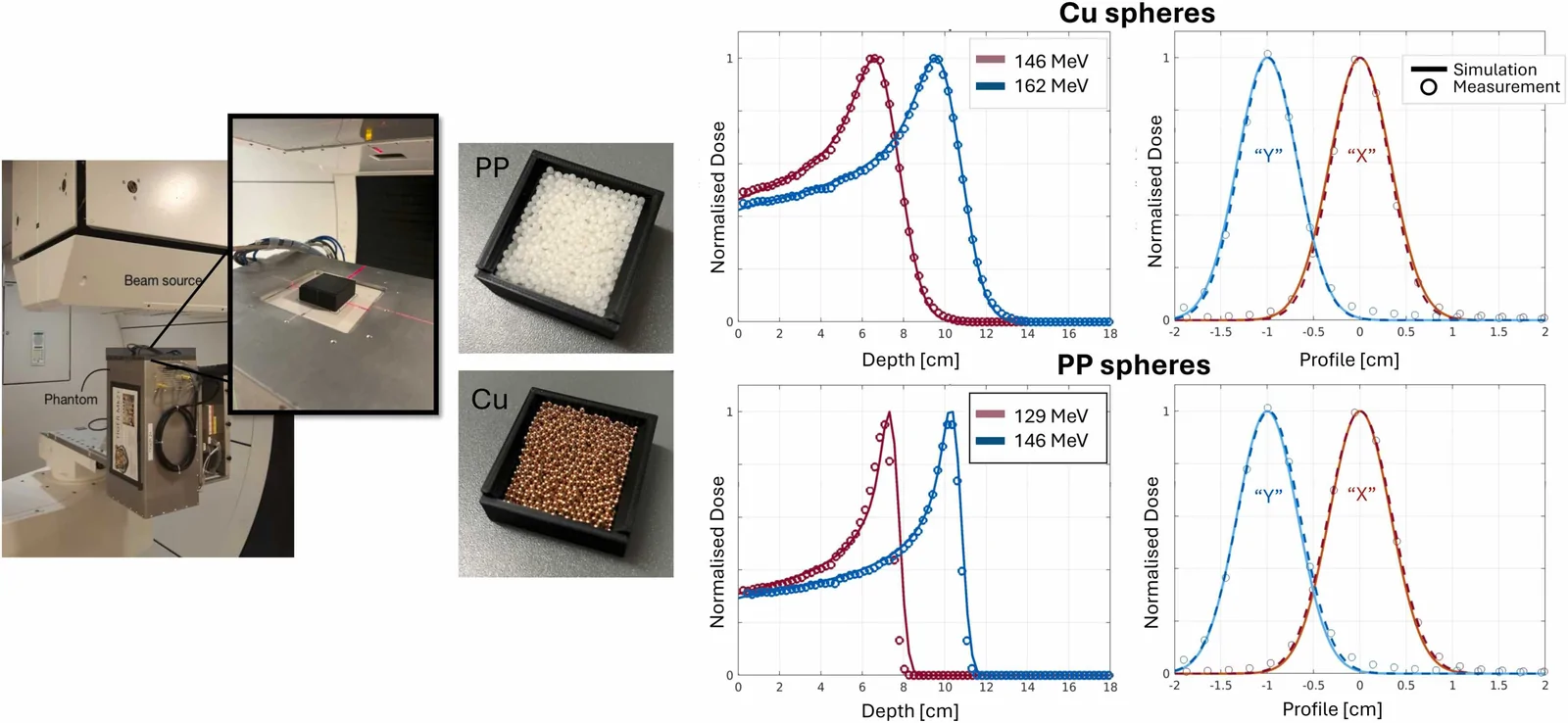
(Left) Experiment setup to measure with the PSI daily QA phantom the DDC and lateral dose profile of the beam passing through simple hollow boxes filled with PP or Cu spheres. (Right) Integral DDC and beam size: comparison between TOPAS simulation (full line) and measurements for a box filled with Cu (top) or PP spheres (bottom).

As a first step of the analysis, we evaluated whether we could correctly reproduce the “eco-friendly” devices with TOPAS MC simulations. With the modified function “pseudo gravity-packing,” we generated one 1.3 cm height box filled with Cu spheres and one 2.3 cm height box filled with PP spheres. We printed a PLA box of the given size, manually filled it with the spheres, and measured the integrated DDC and the beam profile for multiple energies between 100 and 220 MeV (Setup 1). We compared the TOPAS simulation with the measurements for 2 example energies in Figure 2. The range (R80) and beam lateral profile for Cu are within a few percent (relative difference 0.5% and 3%, respectively) and fall within the detector resolution (2.364 mm in depth and 2 mm in the transverse profile). Compared to the pristine Bragg peak, the range is reduced by 4.7 cm (4.8 cm in simulations), and the beam size increases by 2.5% (6% in simulations). For PP, the difference in range is within 5%, with a range decrease of 1.7 cm (1.3 in simulations) compared to the non-degraded DDC. The measured lateral profile aligns with the simulated one, with a relative difference of 10%. The discrepancy between measured and simulated values may be attributed to differences in how the beam lateral profile is modeled in TOPAS, or to an inaccurate specification of the material properties of PLA.

### Collimator performance

We measured and compared the dose profile of a monoenergetic scattered beam after collimating it with an “eco-friendly” collimator and a solid copper collimator (Setup 2). The solid collimator was a copper device of 4 x 4 x 1.5 cm, with an aperture of 1.6 cm in diameter, and the printed PLA hole container was 4 x 4 x 1.7 cm to account for the air space between the spheres. The printing time was less than 20 minutes, and 4 g of PLA filaments were needed.

**Figure 3:**
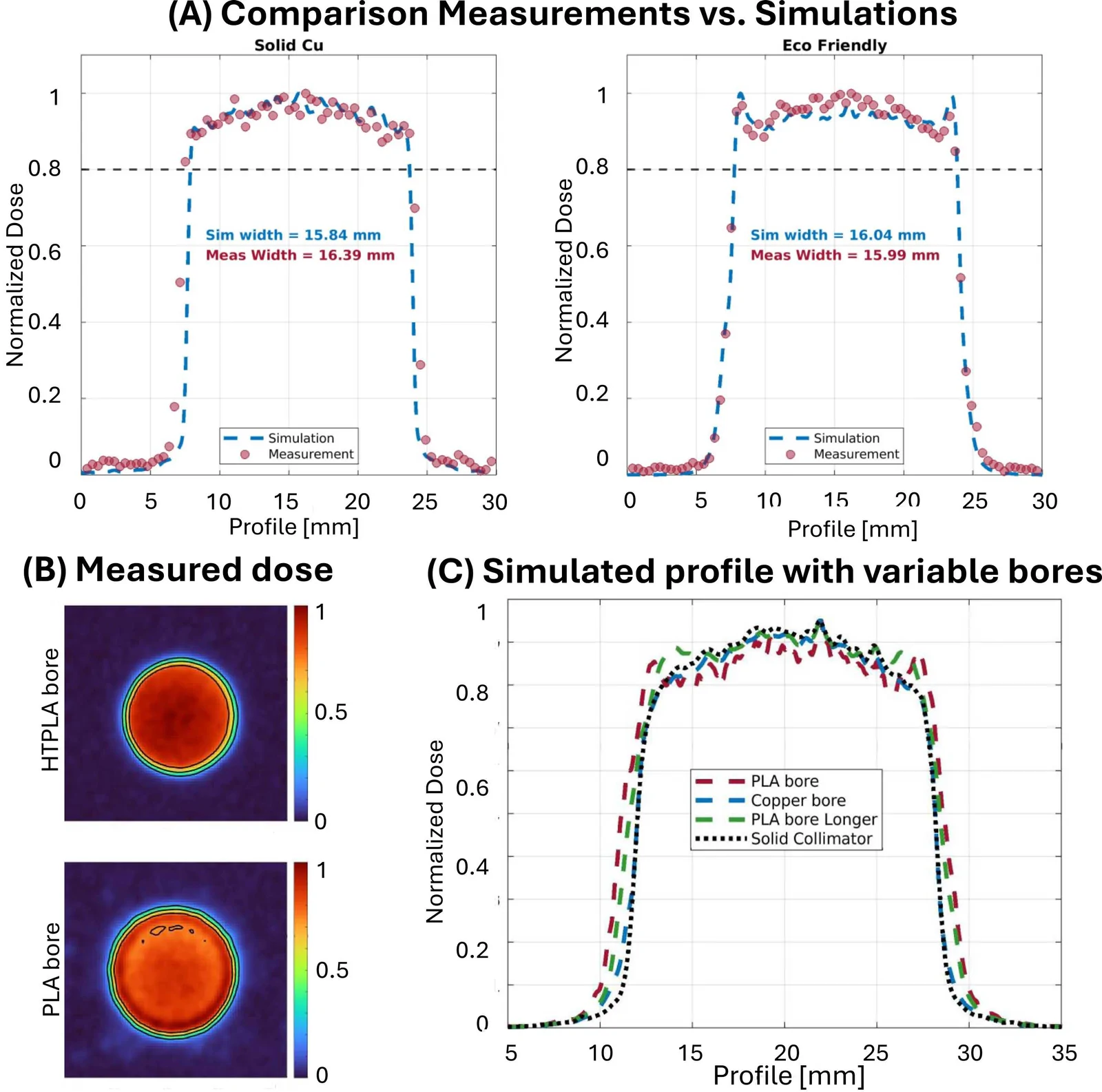
(A) Dose profile: comparison between TOPAS simulation (dashed line) and measurements for scattered beam collimated with an “eco-friendly” collimator (blue) and a solid copper collimator (red). (B) CCD measured 2D dose distribution for an “eco-friendly” collimator with an HTPLA (top) and an PLA (bottom) bore; (C) Simulated dose profile with a PLA bore (red), copper bore (blue), and a longer collimator (green) compared to the corresponding full solid collimator (dotted black).

Figure 3(A) shows a comparison between measurements and simulations for a 90 MeV scattered beam. The difference between the measured and simulated width is within a few percent (1%-3%) for both scenarios. At high energies, we observe that the PLA bore affects the dose distribution, producing a dose halo, see Figure 3(B, PLA bore). This is due to particles not being stopped by the collimator as they pass through the PLA-defined bore. To account for that, we evaluated 2 options. We simulated the PS setup using a collimator in which the bore is defined by copper instead of plastic and by doubling the height of the collimator. In both cases, we reduced the halo dose. However, while the collimator in which the bore is defined by copper effectively prevents all particles from passing through, some particles are still able to pass through the PLA-defined bore despite it being longer. Further, we experimentally tested the superior performance of the copper-bore collimator by 3D-printing the collimator with an HTPLA insert defining the bore, a PLA filament enriched with copper powder, as no full copper filament was available. Results are shown in Figure 3(B, HTPLA bore).

### Target-specific PS delivery

**Figure 4:**
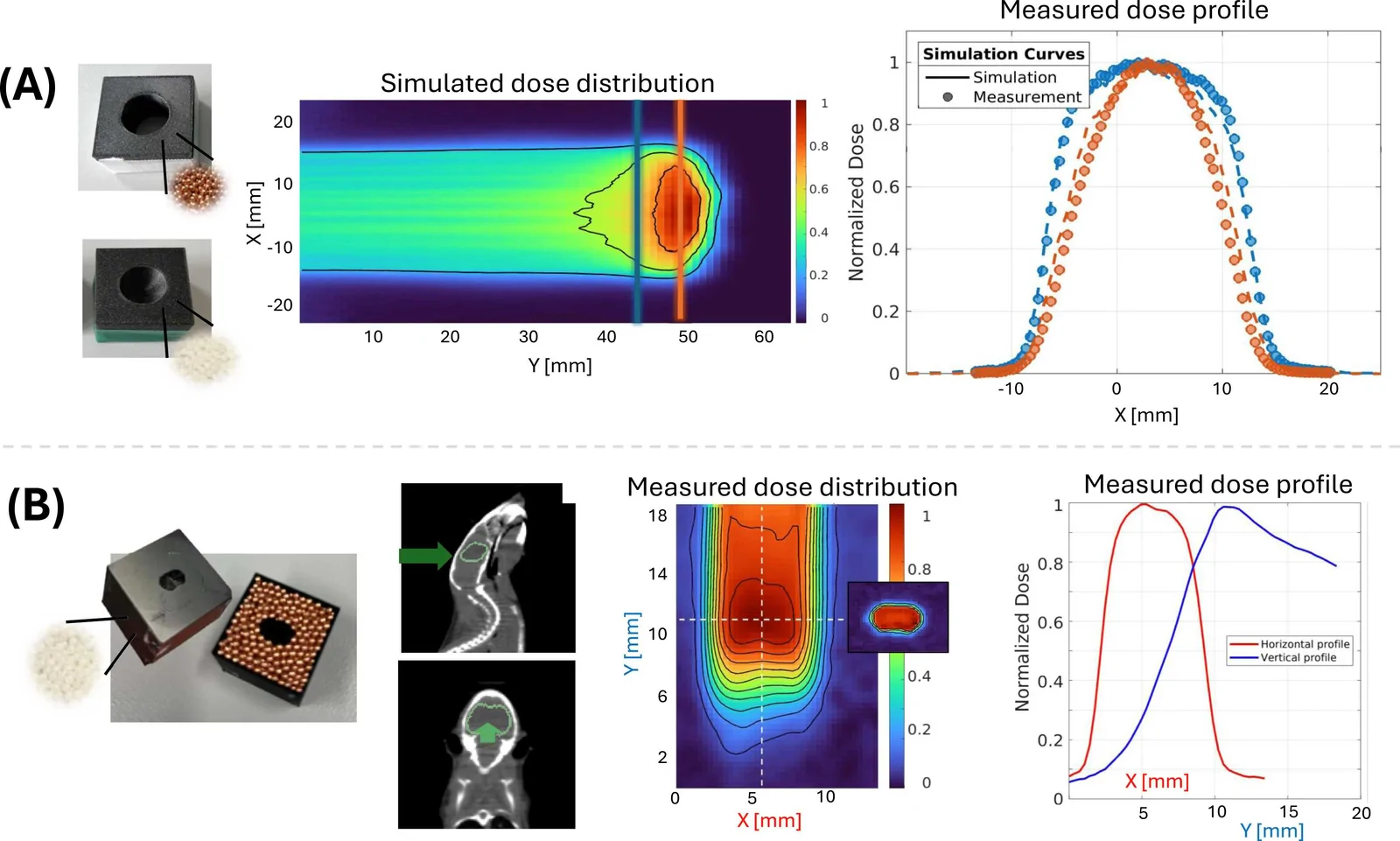
“Eco-friendly” collimator and compensator for (A) a spherical target: (Left) 3D printed collimator and compensator with spheres, (Center) simulated dose profile, (Right) comparison between simulated and measured dose profile (B) a murine brain-like tumor: (Left) 3D printed collimator and compensator with spheres, (Center) mouse CT with contour of the target (volume = 157 mm^3^); (Right) Longitudinal and transversal dose measurements with dose profile.

To test a more experimentally relevant scenario, we designed a collimator and a compensator to irradiate a 2 cm-diameter spherical target. We filled the collimator with Cu spheres and the compensator with PP spheres. The required filament was less than 10 g, and the total printing time was 30 minutes. We simulated and measured (Setup 3) the dose profile for 120 MeV at 2 different depths, as shown in Figure 4(A), and found a mean squared error of less than 1%.

To qualitatively evaluate the feasibility of our method for more complex shapes, we designed an experimental setup to irradiate a murine brain-like tumor. We designed the collimator and compensator to irradiate the targeted area and 3D-printed them. The measured (Setup 3) dose distributions are shown in Figure 4(B).

## Discussion

This study demonstrated the feasibility of hollow 3D-printed compensators and collimators filled with plastic or copper spheres as “eco-friendly” TS devices of small dimensions for small fields PS proton therapy. In addition, a workflow was designed to accurately reproduce them in TOPAS MC, utilizing the modified function available in Porespy^16^ and experimentally validated, which is a necessary step for experiment design. Although the use of 3D-printed cutouts made of plastic shells filled with small balls or pellets is not new in electron beam or conventional radiation therapy,^6^, ^7^, ^18^, ^19^ it has never been tested in proton therapy.

The proposed solution offers a sustainable and cost-effective approach, as the spheres, being the only component requiring an initial 1-time purchase, can be repeatedly used. Containers can be fabricated on demand through 3D printing, utilizing minimal filament due to their hollow structure. This design leads to further savings in both materials and production time, making the approach “eco-friendly.” Additionally, this method enables the fabrication of custom-designed collimator components tailored to each experimental setup, offering greater flexibility compared to reusing legacy hardware that may not be well-suited to current study requirements. The ability to quickly produce customized components improves efficiency and adaptability, especially in the context of small animal irradiations, where precision is crucial. Overall, this strategy not only facilitates more efficient production of PS devices but also helps reduce environmental impact.

Our project aims to conduct a feasibility study to demonstrate the viability of this idea. For clinical applications, it is crucial to ensure quality assurance and reproducibility. Concerns exist regarding the uniformity of sphere distribution, especially since the process of filling the 3D-printed container is performed manually. There may be a need to develop an automated machine for regular use to address this issue.^7^ Before clinical adoption, future work should also include stopping‑power validation across the full clinical energy range, neutron and leakage‑dose characterization, and residual‑activity measurements to ensure radiation‑protection requirements and disposal protocols remain unchanged. Nevertheless, this technique can be investigated for preclinical studies, as highlighted in this study. We found that special attention should be given to the design of the collimator, as the inner cylindrical 3D-printed bore may influence the dose distribution. However, this issue is not a concern when used for low-energy applications such as PS irradiation of mice. One challenge with constructing TS devices is their size. Larger devices require more small spheres, which can lead to increased weight. This issue could be addressed by using a simple box made of copper or plastic that features a predefined square or rectangular opening. We could place the TS device with the 3D-printed shape of our target inside this opening. While each specific target requires a uniquely 3D printed cover for the TS device, the larger copper or plastic holder and the spheres can be reused. Finally, the murine brain-like tumor case was included as a proof-of-concept to show that the proposed approach can be extended to more complex geometries. Within the scope of this technical note, the goal was to demonstrate feasibility rather than provide an exhaustive quantitative analysis. A more detailed anatomical and dosimetric evaluation would be valuable in future work.

In conclusion, our study has demonstrated the effectiveness of an innovative and “eco-friendly” method utilizing 3D-printed cutouts shaped to meet specific target requirements, filled with reusable plastic or copper spheres to create TS shapes. This technique enables rapid production due to its hollow design and the use of reusable materials, such as plastic and copper spheres. By adopting this approach, we reduce waste, lower costs, enhance conformity, and improve the efficiency of TS treatments and experiments.

### Funding

This work was funded by the Swiss National Science Foundation (Grant No. 200882).

## CRediT authorship contribution statement

**Isabella Colizzi:** conceptualization, data curation, formal analysis, validation, investigation, visualization, methodology, writing (original draft), supervision; **Ylva Bornhauser:** data curation, formal analysis, validation, and writing (Review & Editing); **Antony J. Lomax, David Meer, and Serena Psoroula:** methodology, project administration, resources, supervision, writing (Review & Editing), and funding acquisition.

## Declaration of Conflicts of Interest

The authors declare the following financial interests/personal relationships, which may be considered as potential competing interests: Isabella Colizzi reports financial support was provided by the Swiss National Science Foundation. If there are other authors, they declare that they have no known competing financial interests or personal relationships that could have appeared to influence the work reported in this paper.
