# Supplementary material for: A Target-Specific, “Eco-Friendly” Experimental Setup for Small Field Proton Irradiation
Source: Int J Part Ther. 2026 Aug 11;21:101953. doi: 10.1016/j.ijpt.2026.101953 (PMC13499423; doi:10.1016/j.ijpt.2026.101953)
Supplement: Supplementary file 1 [file mmc1.pdf]

Codes

Code x generating the spheres, adapted from pseudogravity package from Porespy

```
logger = logging.getLogger(__name__)
tqdm = get_tqdm()

@njit
def _set_seed(a):
    np.random.seed(a)


def pseudo_gravity_packing(
        im,
        r: int = 5,
        clearance: int = 0,
        axis: int = 0,
        edges: Literal['contained', 'extended'] = 'contained',
        maxiter: int = 1000,
        seed: float = None,
):
    logger.debug(f'Adding spheres of radius {r}')

    if seed is not None:  # Initialize rng so numba sees it
        _set_seed(seed)
        np.random.seed(seed)

    im = np.swapaxes(im, 0, axis)
    im_temp = np.zeros_like(im, dtype=bool)
    r = r
    strel = disk if im.ndim == 2 else ball
    sites = fftmorphology(im == 1, strel=strel(r), mode='erosion')

    inlets = np.zeros_like(im)
    inlets[-(r + 1), ...] = True
    sites = trim_disconnected_blobs(im=sites, inlets=inlets)
    x_min = np.where(sites)[0].min()
    n = None
    for n in tqdm(range(maxiter), **settings.tqdm):
        if im.ndim == 2:
            x, y = np.where(sites[x_min:x_min + 2 * r, ...])
        else:
            x, y, z = np.where(sites[x_min:x_min + 2 * r, ...])
        if len(x) == 0:
```

```
            break
        options = np.where(x == x.min())[0]
        choice = np.random.randint(len(options))
        if im.ndim == 2:
            cen = np.vstack([x[options[choice]] + x_min,
                             y[options[choice]]])
        else:
            cen = np.vstack([x[options[choice]] + x_min,
                             y[options[choice]],
                             z[options[choice]]])
        im_temp = porespy.tools._insert_disks_at_points(im_temp, coords=cen,
                                                        radii=np.array([r]),  #r-clearance
                                                        v=True, overwrite=True)
        sites = porespy.tools._insert_disks_at_points(sites, coords=cen,
                                                      radii=np.array([2 * r + clearance]),  #+clearance
                                                      v=0,
                                                      overwrite=True)
        x_min += x.min()
    logger.debug(f'A total of {n} spheres were added')
    print(f'A total of {n} spheres were added')
    im_temp = np.swapaxes(im_temp, 0, axis)
    return im_temp
```

Function for generating Collimators (with plastic cover)

```
def create_collimator_infill(box_shape, radius, plastic_thickness, plastic_thickness_bottomANDt
op):
    infill = np.ones(box_shape, dtype=bool)
    cylinder_center = [box_shape[1] // 2, box_shape[2] // 2]
    for z in range(box_shape[0]):
        for y in range(box_shape[1]):
            for x in range(box_shape[2]):
                if z > plastic_thickness_bottomANDtop or z <= (box_shape[0] - plastic_thickness_bott
omANDtop):
                    if np.sqrt((y - cylinder_center[0]) ** 2 + (x - cylinder_center[1]) ** 2) < (
                            cylinder_radius + plastic_thickness):
                        infill[z, y, x] = False  # Set the cylindrical region to False

    shape = pseudo_gravity_packing(im=infill, r=radius, clearance=0, maxiter=18000)
    return shape

def create_collimator_cover(box_shape, cylinder_radius, plastic_thickness, plastic_thickness_b
ottomANDtop):
    cover = np.ones(box_shape, dtype=bool)
    cylinder_center = [box_shape[1] // 2, box_shape[2] // 2]
    for z in range(box_shape[0]):
        for y in range(box_shape[1]):
```

```
            for x in range(box_shape[2]):
                # Calculate the distance from the center of the cylindrical hole
                distance_from_center = np.sqrt((y - cylinder_center[0]) ** 2 + (x - cylinder_center[1])
** 2)

                # Set the cylindrical hole throughout the entire box height (including top and bottom)
                if distance_from_center < cylinder_radius:  # Inside the cylindrical hole (hollow)
                    cover[z, y, x] = False  # Empty inside the cylinder

                # Cylindrical wall with a thickness around the hole
                elif cylinder_radius <= distance_from_center < (
                        cylinder_radius + plastic_thickness):  # Cylindrical wall
                    cover[z, y, x] = True  # Solid cylindrical wall

                # For top and bottom layers (solid with a thickness of plastic_thickness_bottomANDto
p), exclude the hole
                elif z < plastic_thickness_bottomANDtop or z >= (box_shape[0] - plastic_thickness_bo
ttomANDtop):
                    if distance_from_center >= (cylinder_radius + plastic_thickness):  # Outside the hol
e
                        cover[z, y, x] = True  # Solid top/bottom layer

                else:
                    cover[z, y, x] = False  # Everything else is hollow
    return cover
```

Function for generating Compensators with spherical shape (with plastic cover)

```
def create_compensator_infill(box_shape, radius, sphere_radius, plastic_thickness):
    box = np.ones(box_shape, dtype=bool)
    half_sphere = np.zeros(box_shape, dtype=bool)
    sphere_center = [0, box_shape[1] // 2, box_shape[2] // 2]

    for z in range(box_shape[0]):
        for y in range(box_shape[1]):
            for x in range(box_shape[2]):
                # Calculate the distance from the sphere center
                distance = np.sqrt(
                    (z - sphere_center[0]) ** 2 + (y - sphere_center[1]) ** 2 + (x - sphere_center[2]) **
2)
                # Check if the point is within the half-sphere (and ensure the peak is inside the box)
                if distance < (sphere_radius - plastic_thickness) and z < (sphere_radius - plastic_thick
ness):
                    half_sphere[z, y, x] = True  # Set the spherical region to True

    # Subtract the half-sphere from the box
```

```
    image = box & ~half_sphere
    compensator = pseudo_gravity_packing(im=image, r=radius, clearance=0, maxiter=20000)
    return compensator


def create_compensator_cover(box_shape, sphere_radius, plastic_thickness):
    half_sphere_cover = np.zeros(box_shape, dtype=bool)
    sphere_center = [0, box_shape[1] // 2, box_shape[2] // 2]
    for z in range(box_shape[0]):
        for y in range(box_shape[1]):
            for x in range(box_shape[2]):
                distance = np.sqrt(
                    (z - sphere_center[0]) ** 2 + (y - sphere_center[1]) ** 2 + (x - sphere_center[2]) **
2)
                # Create the spherical shell with a thickness
                if sphere_radius <= distance < (sphere_radius + plastic_thickness) and z < (
                        sphere_radius + plastic_thickness):
                    half_sphere_cover[z, y, x] = True  # Set the spherical region to True

                # Add contour thickness only to the lower z boundary
                if z > box_shape[0] - plastic_thickness:
                    half_sphere_cover[z, y, x] = True  # Solid bottom layer
    return half_sphere_cover
```

How to run and save?

```
import numpy as np
import matplotlib.pyplot as plt
import porespy
from scipy.ndimage import distance_transform_edt as edt
from porespy.tools import _insert_disks_at_points, get_tqdm
from porespy.filters import trim_disconnected_blobs, fftmorphology
from numba import njit
from typing import Literal
import logging
import scipy.ndimage as spim
from edt import edt
from skimage.morphology import disk, ball
from porespy import settings
import trimesh
import matplotlib.pyplot as plt

if __name__ == "__main__":
    box_shape = [170, 390, 390]  # Depth, Height, Width
    image = np.ones(box_shape, dtype=bool)
    cover = np.ones(box_shape, dtype=bool)
```

```
    sphere_radius = 100
    cylinder_radius = 160 / 2
    plastic_thickness_cylinder = 50
    plastic_thickness_bottomANDtop = 10
    cylinder_center = [box_shape[1] // 2, box_shape[2] // 2]

    #obj = create_collimator_infill(box_shape, sphere_radius, cylinder_radius, plastic_thickness_c
ylinder, plastic_thickness_bottomANDtop)
    #obj = create_collimator_cover(box_shape, sphere_radius, cylinder_radius, plastic_thickness_
cylinder, plastic_thickness_bottomANDtop)
    #obj = create_collimatpr_to_print(box_shape, sphere_radius, cylinder_radius, plastic_thicknes
s_cylinder, plastic_thickness_bottomANDtop)
    #obj = create_compensator_infill(box_shape, sphere_radius, sphere_radius, plastic_thickness
_bottomANDtop)
    obj = create_compensator_cover(box_shape, sphere_radius, sphere_radius, plastic_thickness
_bottomANDtop)

    mesh = trimesh.voxel.VoxelGrid(obj).marching_cubes
    mesh.export('compensator_cover.stl', file_type='stl')
    print('STL file exported successfully')
```

Code for generating and plotting simple simple boxes (+integral density):

```
    shape = [245, 100, 100] # Depth, Height, Width
    image = np.ones(shape, dtype=bool)

    im = pseudo_gravity_packing(im=np.ones(shape, dtype=bool),r=12, clearance=0,edges='co
ntained', seed=3, maxiter=3500)
    int_array_2d_1 = np.sum(im, axis=(1, 2)) # integral denisty

    fig, ax = plt.subplots(1, 2, figsize=(10, 6))
    ax[0].imshow(porespy.visualization.show_3D(~im))
    ax[1].plot(int_array_2d_1)

    mesh = trimesh.voxel.VoxelGrid(image).marching_cubes
    mesh.export('simple_box_full.stl')
    print('STL file exported successfull')
```

Code for generating topas input file (with topas components)

```
import numpy as np
import matplotlib.pyplot as plt
import porespy
from scipy.ndimage import distance_transform_edt as edt
```

```
from porespy.tools import _insert_disks_at_points, get_tqdm
from porespy.filters import trim_disconnected_blobs, fftmorphology
from numba import njit
from typing import Literal
import logging
import scipy.ndimage as spim
from edt import edt
from skimage.morphology import disk, ball
from porespy import settings
import trimesh
import matplotlib.pyplot as plt

tqdm = get_tqdm()

@njit
def _set_seed(a):
    np.random.seed(a)


def pseudo_gravity_packing(im, r: int = 5, clearance: int = 0, axis: int = 0,
                           edges: Literal['contained', 'extended'] = 'contained', maxiter: int = 1000,
                           seed: float = None):
    if seed is not None:  # Initialize rng so numba sees it
        _set_seed(seed)
        np.random.seed(seed)

    im = np.swapaxes(im, 0, axis)
    im_temp = np.zeros_like(im, dtype=bool)
    r = r
    strel = disk if im.ndim == 2 else ball
    sites = fftmorphology(im == 1, strel=strel(r), mode='erosion')

    inlets = np.zeros_like(im)
    inlets[-(r + 1), ...] = True
    sites = trim_disconnected_blobs(im=sites, inlets=inlets)
    x_min = np.where(sites)[0].min()

    coords_list = []  # Initialize list to store coordinates of spheres

    n = None
    for n in tqdm(range(maxiter), **settings.tqdm):
        if im.ndim == 2:
            x, y = np.where(sites[x_min:x_min + 2 * r, ...])
        else:
            x, y, z = np.where(sites[x_min:x_min + 2 * r, ...])
        if len(x) == 0:
            break
        options = np.where(x == x.min())[0]
```

```
        choice = np.random.randint(len(options))
        if im.ndim == 2:
            cen = np.vstack([x[options[choice]] + x_min, y[options[choice]]])
            coords_list.append([cen[0][0], cen[1][0]])  # Append 2D coordinates
        else:
            cen = np.vstack([x[options[choice]] + x_min, y[options[choice]], z[options[choice]]])
            coords_list.append([cen[0][0], cen[1][0], cen[2][0]])  # Append 3D coordinates

        im_temp = porespy.tools._insert_disks_at_points(im_temp, coords=cen,
                                                        radii=np.array([r]),  # r-clearance
                                                        v=True, overwrite=True)
        sites = porespy.tools._insert_disks_at_points(sites, coords=cen,
                                                      radii=np.array([2 * r + clearance]),  # +clearance
                                                      v=0, overwrite=True)
        x_min += x.min()

    print(f'A total of {n} spheres were added')
    im_temp = np.swapaxes(im_temp, 0, axis)

    return im_temp, coords_list, n  # Return the packed image and the list of coordinates

def create_collimator_infill(box_shape, radius, cylinder_radius, plastic_thickness, plastic_thickn
ess_bottomANDtop):
    infill = np.ones(box_shape, dtype=bool)
    cylinder_center = [box_shape[1] // 2, box_shape[2] // 2]
    for z in range(box_shape[0]):
        for y in range(box_shape[1]):
            for x in range(box_shape[2]):
                if z > plastic_thickness_bottomANDtop or z <= (box_shape[0] - plastic_thickness_bott
omANDtop):
                    if np.sqrt((y - cylinder_center[0]) ** 2 + (x - cylinder_center[1]) ** 2) < (
                            cylinder_radius + plastic_thickness):
                        infill[z, y, x] = False  # Set the cylindrical region to False

    im, coord, n_spheres = pseudo_gravity_packing(im=infill, r=radius, clearance=0, maxiter=180
00)
    return im, coord, n_spheres


def create_collimator_conic_infill(box_shape, radius, cylinder_radius_IN, cylinder_radius_OUT, p
lastic_thickness,
                             plastic_thickness_bottomANDtop):
    infill = np.ones(box_shape, dtype=bool)
    cylinder_center = [box_shape[1] // 2, box_shape[2] // 2]
    radius_step = (cylinder_radius_OUT - cylinder_radius_IN) / box_shape[0]
    for z in range(box_shape[0]):
        current_radius = cylinder_radius_IN + z * radius_step
        for y in range(box_shape[1]):
```

```
            for x in range(box_shape[2]):
                if plastic_thickness_bottomANDtop < z <= (box_shape[0] - plastic_thickness_bottomA
NDtop):
                    distance_from_center = np.sqrt((y - cylinder_center[0]) ** 2 + (x - cylinder_center
[1]) ** 2)
                    if distance_from_center < (current_radius + plastic_thickness):
                        infill[z, y, x] = False

    im, coord, n_spheres =  pseudo_gravity_packing(im=infill, r=radius, clearance=0, maxiter=18
000)

    return im, coord, n_spheres

def create_compensator_infill(box_shape,radius, sphere_radius, plastic_thickness_bottomANDt
op):
    box = np.ones(box_shape, dtype=bool)
    half_sphere = np.zeros(box_shape, dtype=bool)
    sphere_center = [0, box_shape[1] // 2, box_shape[2] // 2]

    for z in range(box_shape[0]):
        for y in range(box_shape[1]):
            for x in range(box_shape[2]):
                # Calculate the distance from the sphere center
                distance = np.sqrt(
                    (z - sphere_center[0]) ** 2 + (y - sphere_center[1]) ** 2 + (x - sphere_center[2]) **
2)
                # Check if the point is within the half-sphere (and ensure the peak is inside the box)
                if distance < (sphere_radius - plastic_thickness_bottomANDtop) and z < (sphere_radi
us - plastic_thickness_bottomANDtop):
                    half_sphere[z, y, x] = True  # Set the spherical region to True

    # Subtract the half-sphere from the box
    infill = box & ~half_sphere
    im, coord, n_spheres =  pseudo_gravity_packing(im=infill, r=radius, clearance=0, maxiter=18
000)

    return im, coord, n_spheres


def create_spheres_txt(coord, sphere_radius, output_filename="spheres_output.txt"):
    # Header section
    txt_content = """########################################
# Spheres Group - Collimator
########################################

s:Ge/CollimatorGroup/Parent = "World"
s:Ge/CollimatorGroup/Type   = "Group"
dc:Ge/CollimatorGroup/RotX = 0 deg
```

```
dc:Ge/CollimatorGroup/RotY = 0 deg
dc:Ge/CollimatorGroup/RotZ = 0 deg
dc:Ge/CollimatorGroup/TransX = -1.8 cm
dc:Ge/CollimatorGroup/TransY = -2 cm
dc:Ge/CollimatorGroup/TransZ = -2 cm

########################################
# Spheres
########################################

"""

    # Iterate over the coordinates to add each sphere
    for i, (x, y, z) in enumerate(coord):
        sphere_num = i + 1
        # Create the sphere section for the current sphere
        sphere_section = f"""s:Ge/Sphere{sphere_num}/Type                = "TsSphere"
s:Ge/Sphere{sphere_num}/Parent              = "CollimatorGroup"
s:Ge/Sphere{sphere_num}/Material            = "Copper"
d:Ge/Sphere{sphere_num}/TransX              = {x / 10:.1f} mm
d:Ge/Sphere{sphere_num}/TransY              = {y / 10:.1f} mm
d:Ge/Sphere{sphere_num}/TransZ              = {z / 10:.1f} mm
d:Ge/Sphere{sphere_num}/RotX                = 0 deg
d:Ge/Sphere{sphere_num}/RotY                = 0 deg
d:Ge/Sphere{sphere_num}/RotZ                = 0 deg
d:Ge/Sphere{sphere_num}/RMin                = 0 mm
d:Ge/Sphere{sphere_num}/RMax                = {sphere_radius / 10:.1f} mm
d:Ge/Sphere{sphere_num}/SPhi                = 0 deg
d:Ge/Sphere{sphere_num}/DPhi                = 360 deg
d:Ge/Sphere{sphere_num}/STheta              = 0 deg
d:Ge/Sphere{sphere_num}/DTheta              = 180 deg
s:Ge/Sphere{sphere_num}/DrawingStyle        = "FullWireFrame"
s:Ge/Sphere{sphere_num}/Color               = "grass"

"""
        # Append each sphere's section to the full content
        txt_content += sphere_section

    # Write the final content to a file
    with open(output_filename, "w") as file:
        file.write(txt_content)
    print(f"File '{output_filename}' created successfully!")



if __name__ == "__main__":
    box_shape = [170, 400, 400]  # Depth, Height, Width
    image = np.ones(box_shape, dtype=bool)
    cover = np.ones(box_shape, dtype=bool)
```

```
    sphere_radius = 10
    sphere_radius_PP = 12
    concavity_radius = 160 / 2
    cylinder_radius = 160 / 2
    cylinder_radius_out = 200 / 2
    plastic_thickness_cylinder = 5
    plastic_thickness_bottomANDtop = 5
    cylinder_center = [box_shape[1] // 2, box_shape[2] // 2]

    im, coord, n_spheres = create_collimator_infill(box_shape, sphere_radius, cylinder_radius, pla
stic_thickness_cylinder, plastic_thickness_bottomANDtop)
    #im, coord, n_spheres = create_compensator_infill(box_shape,sphere_radius_PP, concavity_r
adius, plastic_thickness_bottomANDtop)
    #im, coord, n_spheres = create_collimator_conic_infill(box_shape, sphere_radius, cylinder_ra
dius,cylinder_radius_out,
                                             # plastic_thickness_cylinder, plastic_thickness_bottomANDtop)


    # Print the first few coordinates
    print("First 10 coordinates where spheres are placed:")
    for i, c in enumerate(coord[:10]):  # Print first 10 coordinates
        print(f"Sphere {i + 1}: {c}")

    # Print total number of spheres added
    print(f"Total number of spheres added: {n_spheres}")

    create_spheres_txt(coord, sphere_radius, output_filename="collimator170×390×390_d160_To
pas_inputfile.txt")
```

generate code from stl file

```

import open3d as o3d

def stl_to_voxel(stl_file, voxel_resolution):
    """Convert an STL file into a voxelized 3D array using Open3D."""

    # Load STL file as a triangle mesh
    mesh = o3d.io.read_triangle_mesh(stl_file)

    # Ensure the mesh has vertex normals
    mesh.compute_vertex_normals()

    # Create a voxel grid from the mesh
    voxel_grid = o3d.geometry.VoxelGrid.create_from_triangle_mesh(mesh, voxel_size=voxel_res
olution)

```

```
    # Get voxel centers
    voxels = np.array([v.grid_index for v in voxel_grid.get_voxels()])

    # Compute grid shape
    if voxels.size == 0:
        raise ValueError("Voxelization failed: No voxels were generated. Check STL file and resolut
ion.")

    min_bounds = voxels.min(axis=0)
    max_bounds = voxels.max(axis=0)
    grid_shape = max_bounds - min_bounds + 1  # Add 1 to include edges

    # Create a voxel array
    voxel_array = np.zeros(grid_shape, dtype=bool)

    # Convert voxel positions to the array indices
    for v in voxels:
        voxel_array[tuple(v - min_bounds)] = True  # Shift to start from (0,0,0)

    # Fill holes to ensure a solid object
    voxel_array = binary_fill_holes(voxel_array)

    return voxel_array, voxel_grid

def create_collimator_infill_from_stl(stl_file, voxel_resolution, radius):
    """Generate the infill within the STL-defined collimator."""

    stl_voxel, voxel_grid = stl_to_voxel(stl_file, voxel_resolution)
    # o3d.visualization.draw_geometries([voxel_grid])

    im, coord, n_spheres = pseudo_gravity_packing(im=stl_voxel, r=radius, clearance=0, maxiter
=18000)

    return im, coord, n_spheres



if __name__ == "__main__":

    #image = np.ones(box_shape, dtype=bool)
    #cover = np.ones(box_shape, dtype=bool)

    sphere_radius = 10
    sphere_radius_PP = 12
    concavity_radius = 160 / 2
    cylinder_radius = 160 / 2
    cylinder_radius_out = 200 / 2
    plastic_thickness_cylinder = 5
    plastic_thickness_bottomANDtop = 0
```

```
    im, coord, n_spheres = create_collimator_infill_from_stl("collimatorGTV_small.stl", 0.1, sphere_radius)
    #plot_voxel_array(im)
    create_spheres_txt(coord, sphere_radius, output_filename="collimatorSmall_GTV_topasInput.txt")
```
