# Supplementary material for: A Target-Specific, “Eco-Friendly” Experimental Setup for Small Field Proton Irradiation
Source: Int J Part Ther. 2026 Aug 11;21:101953. doi: 10.1016/j.ijpt.2026.101953 (PMC13499423; doi:10.1016/j.ijpt.2026.101953)
Supplement: Supplementary file 2 [file mmc2.pdf]

```
Ge/World/Material = "Air"
Ge/World/HLX=1.5 m
Ge/World/HLY=1.5 m
Ge/World/HLZ=1.5 m

########################### Origin of the beam ###########################

s:Ge/SourceOrigin/Parent="World"
s:Ge/SourceOrigin/Type="Group"
dc:Ge/SourceOrigin/TransX= -47.8 cm
dc:Ge/SourceOrigin/TransY= 0 mm
dc:Ge/SourceOrigin/TransZ= 0 mm
dc:Ge/SourceOrigin/RotX= 0.0 deg
dc:Ge/SourceOrigin/RotY= -90.00 deg
dc:Ge/SourceOrigin/RotZ= 0. deg
b:Ge/CheckForOverlaps = "False"

s:So/Source/Component = "SourceOrigin"
s:So/Source/BeamParticle = "proton"
s:So/Source/Type           = "emittance"
s:So/Source/Distribution   = "BiGaussian"
i:So/Source/NumberOfHistoriesInRun = 100 #number of particles
Ge/QuitIfOverlapDetected = "false"

i:Ts/ShowHistoryCountAtInterval = 0
i:Ts/NumberOfThreads = 0
#b:Ts/PauseBeforeQuit = "True"
i:Ts/MaxInterruptedHistories = 2147483647


Ge/CheckForUnusedComponents = "False"
b:Ge/CheckInsideEnvelopesForOverlaps = "False"
b:Ph/ListProcesses = "False"
i:Ts/RunVerbosity = 0
i:Ts/EventVerbosity = 0
i:Ts/TrackingVerbosity = 0
i:Ts/SequenceVerbosity = 0
i:Ts/InterruptedHistoryMaxReports = 0

########################### choose the energy (example 120MeV)
###########################


d:So/Source/BeamEnergy = 120047.0 keV
u:So/Source/BeamEnergySpread = 0.684655
d:So/Source/SigmaX = 0.330594 cm
u:So/Source/SigmaXPrime = 0.005381
u:So/Source/CorrelationX = -0.20987
d:So/Source/SigmaY = 0.284945 cm
u:So/Source/SigmaYPrime = 0.005486
u:So/Source/CorrelationY = 0.02029


########################### materials list ###########################

sv:Ma/Water/Components = 2 "Hydrogen" "Oxygen"
uv:Ma/Water/Fractions = 2 0.111894 0.888106
d:Ma/Water/Density = 1 g/cm3
d:Ma/Water/MeanExcitationEnergy = 75.0 eV
s:Ma/Water/DefaultColor = "indigo"

sv:Ma/Air/Components = 4 "Carbon" "Nitrogen" "Oxygen" "Argon"
```

```
uv:Ma/Air/Fractions = 4 0.000124 0.755268 0.231781 0.012827
d:Ma/Air/Density = 1.20484 mg/cm3
d:Ma/Air/MeanExcitationEnergy = 85.7 eV
s:Ma/Air/DefaultColor = "lightblue"

# important: we are using an infill of 50%, so probaly the density is 50% of that
sv:Ma/PLA/Components = 3 "Carbon" "Hydrogen" "Oxygen"
uv:Ma/PLA/Fractions = 3 0.5 0.0556 0.4444
d:Ma/PLA/Density = 1.24 g/cm3 # 50% of 1.24 g/cm3 (?)
d:Ma/PLA/MeanExcitationEnergy = 73 eV
s:Ma/PLA/DefaultColor = "lightblue"

# we will need to find the composition of PP (check pstar database, probably similar
to Polyethylene?)
sv:Ma/Polypropylene/Components = 2 "Carbon" "Hydrogen"
uv:Ma/Polypropylene/Fractions = 2 0.856289 0.143711
d:Ma/Polypropylene/Density = 1.24 g/cm3
d:Ma/Polypropylene/MeanExcitationEnergy = 56.5 eV
s:Ma/Polypropylene/DefaultColor = "lightblue"

sv:Ma/Copper/Components = 1 "Copper"
uv:Ma/Copper/Fractions = 1 1.0
d:Ma/Copper/Density = 8.96 g/cm3
d:Ma/Copper/MeanExcitationEnergy = 322 eV
s:Ma/Copper/DefaultColor = "orange"

sv:Ma/Aluminum/Components = 1 "Aluminum"
uv:Ma/Aluminum/Fractions = 1 1.0
d:Ma/Aluminum/Density = 2.699 g/cm3
d:Ma/Aluminum/MeanExcitationEnergy = 166 eV
s:Ma/Aluminum/DefaultColor = "silver"


sv:Ma/PMMA/Components = 3 "Hydrogen" "Carbon" "Oxygen"
uv:Ma/PMMA/Fractions = 3 0.080538 0.599848 0.319614
d:Ma/PMMA/Density = 1.19 g/cm3
d:Ma/PMMA/MeanExcitationEnergy = 74 eV
s:Ma/PMMA/DefaultColor = "lightblue"

sv:Ma/Carbon/Components = 1 "Carbon"
uv:Ma/Carbon/Fractions = 1 1.0
d:Ma/Carbon/Density = 1.867 g/cm3
d:Ma/Carbon/MeanExcitationEnergy = 78 eV
s:Ma/Carbon/DefaultColor = "green"

#################################################### geometry
#####################################################


########################## Set the preabsorber (if used) ##########################
s:Ge/PMMABox/Type="TsBox"
s:Ge/PMMABox/Material="Carbon" #CHANGE
s:Ge/PMMABox/Parent="World"
d:Ge/PMMABox/HLX= 10 cm
d:Ge/PMMABox/HLY= 10 cm
d:Ge/PMMABox/HLZ=   1.25 cm
dc:Ge/PMMABox/TransZ = 0 cm
dc:Ge/PMMABox/TransY = 0 cm
dc:Ge/PMMABox/TransX = -45.55  cm
dc:Ge/PMMABox/RotX = 0. deg
dc:Ge/PMMABox/RotY = -90 deg
dc:Ge/PMMABox/RotZ = 0 deg
```

```
s:Ge/PMMABox2/Type="TsBox"
s:Ge/PMMABox2/Material="PMMA" #CHANGE
s:Ge/PMMABox2/Parent="World"
d:Ge/PMMABox2/HLX= 10 cm
d:Ge/PMMABox2/HLY= 10 cm
d:Ge/PMMABox2/HLZ=   0.5 cm
dc:Ge/PMMABox2/TransZ = 0 cm
dc:Ge/PMMABox2/TransY = 0 cm
dc:Ge/PMMABox2/TransX = -5  cm
dc:Ge/PMMABox2/RotX = 0. deg
dc:Ge/PMMABox2/RotY = -90 deg
dc:Ge/PMMABox2/RotZ = 0 deg

########################### Set the extra copper as collimator
##########################
s:Ge/CopperBox1/Type="TsBox"
s:Ge/CopperBox1/Material="Copper"
s:Ge/CopperBox1/Parent="World"
dc:Ge/CopperBox1/HLX= 4 cm
dc:Ge/CopperBox1/HLY= 8 cm
dc:Ge/CopperBox1/HLZ=  0.5 cm
dc:Ge/CopperBox1/TransZ = 6 cm
dc:Ge/CopperBox1/TransY = 0 cm
dc:Ge/CopperBox1/TransX = -0.5  cm
dc:Ge/CopperBox1/RotX = 0. deg
dc:Ge/CopperBox1/RotY = -90 deg
dc:Ge/CopperBox1/RotZ = 0 deg

# Set the extra copper
s:Ge/CopperBox2/Type="TsBox"
s:Ge/CopperBox2/Material="Copper"
s:Ge/CopperBox2/Parent="World"
dc:Ge/CopperBox2/HLX= 2 cm
dc:Ge/CopperBox2/HLY= 3 cm
dc:Ge/CopperBox2/HLZ=  0.5 cm
dc:Ge/CopperBox2/TransZ = 0 cm
dc:Ge/CopperBox2/TransY = -5 cm
dc:Ge/CopperBox2/TransX = -0.5 cm
dc:Ge/CopperBox2/RotX = 0. deg
dc:Ge/CopperBox2/RotY = -90 deg
dc:Ge/CopperBox2/RotZ = 0 deg


# Set the extra copper
s:Ge/CopperBox3/Type="TsBox"
s:Ge/CopperBox3/Material="Copper"
s:Ge/CopperBox3/Parent="World"
dc:Ge/CopperBox3/HLX= 4 cm
dc:Ge/CopperBox3/HLY= 8 cm
dc:Ge/CopperBox3/HLZ=  0.5 cm
dc:Ge/CopperBox3/TransZ = -6 cm
dc:Ge/CopperBox3/TransY = 0 cm
dc:Ge/CopperBox3/TransX = -0.5  cm
dc:Ge/CopperBox3/RotX = 0. deg
dc:Ge/CopperBox3/RotY = -90 deg
dc:Ge/CopperBox3/RotZ = 0 deg

# Set the extra copper
s:Ge/CopperBox4/Type="TsBox"
s:Ge/CopperBox4/Material="Copper"
s:Ge/CopperBox4/Parent="World"
```

```
dc:Ge/CopperBox4/HLX= 2 cm
dc:Ge/CopperBox4/HLY= 3 cm
dc:Ge/CopperBox4/HLZ=  0.5 cm
dc:Ge/CopperBox4/TransZ = 0 cm
dc:Ge/CopperBox4/TransY = 5 cm
dc:Ge/CopperBox4/TransX = -0.5   cm
dc:Ge/CopperBox4/RotX = 0. deg
dc:Ge/CopperBox4/RotY = -90 deg
dc:Ge/CopperBox4/RotZ = 0 deg

########################## collimator hallow component ##########################

# Cylinder
s:Ge/Cylinder/Type               = "TsCylinder"
s:Ge/Cylinder/Parent             = "World"
s:Ge/Cylinder/Material           = "PLA"
dc:Ge/Cylinder/TransX             = -3 cm
dc:Ge/Cylinder/TransY             = 0 cm
dc:Ge/Cylinder/TransZ             = 0 cm
dc:Ge/Cylinder/RotX               = 0 deg
dc:Ge/Cylinder/RotY               = -90 deg
dc:Ge/Cylinder/RotZ               = 0 deg
d:Ge/Cylinder/RMin               = 10 mm
d:Ge/Cylinder/RMax               = 10.5 mm
d:Ge/Cylinder/HL                 = 1 cm
dc:Ge/Cylinder/SPhi               = 0. rad
dc:Ge/Cylinder/DPhi               = 360 deg
s:Ge/Cylinder/Color              = "lightblue"
s:Ge/Cylinder/DrawingStyle       = "FullWireFrame"

# Set the box top and bottom
s:Ge/CylinderTop/Type               = "TsCylinder"
s:Ge/CylinderTop/Parent             = "World"
s:Ge/CylinderTop/Material           = "PLA"
dc:Ge/CylinderTop/TransX             = -4.2 cm
dc:Ge/CylinderTop/TransY             = 0 cm
dc:Ge/CylinderTop/TransZ             = 0 cm
dc:Ge/CylinderTop/RotX               = 0 deg
dc:Ge/CylinderTop/RotY               = -90 deg
dc:Ge/CylinderTop/RotZ               = 0 deg
d:Ge/CylinderTop/RMin               = 10 mm
d:Ge/CylinderTop/RMax               = 20 mm
d:Ge/CylinderTop/HL                 = 2 mm
dc:Ge/CylinderTop/SPhi               = 0. rad
dc:Ge/CylinderTop/DPhi               = 360 deg
s:Ge/CylinderTop/Color              = "lightblue"
s:Ge/CylinderTop/DrawingStyle       = "FullWireFrame"


### material in the box
s:Ge/MyPartFromCAD/Type = "TsCAD"
s:Ge/MyPartFromCAD/Parent = "World"
s:Ge/MyPartFromCAD/Material = "PLA"
dc:Ge/MyPartFromCAD/TransX = -2 cm  #here
dc:Ge/MyPartFromCAD/TransY = -2 cm
dc:Ge/MyPartFromCAD/TransZ = -2 cm
dc:Ge/MyPartFromCAD/RotX = 0.0 deg
dc:Ge/MyPartFromCAD/RotY = 0.0 deg
dc:Ge/MyPartFromCAD/RotZ = -0. rad
s:Ge/MyPartFromCAD/DrawingStyle = "Solid"
s:Ge/MyPartFromCAD/Color              = "green"
```

```
######################### compensator hallow component #########################

## CAD-specific parameters:
s:Ge/MyPartFromCAD/InputFile = "compensator_cover"
s:Ge/MyPartFromCAD/FileFormat = "stl"
d:Ge/MyPartFromCAD/Units = 0.1 mm

######################### compensator + collimator list of TsSpheres (created with
pyhton scripts) #########################

includeFile =  compensator_spheres.txt
includeFile = collimator_spheres.txt

######################### scoring ##########################
s:Ge/Detector/Type                   = "TsBox"
s:Ge/Detector/Parent                 = "World"
s:Ge/Detector/Material                = "PMMA"
d:Ge/Detector/TransX                 = 4 cm
d:Ge/Detector/TransY                 = 0 cm
d:Ge/Detector/TransZ                 = 0 cm
d:Ge/Detector/RotX                   = 0 deg
d:Ge/Detector/RotY                   = -90 deg
d:Ge/Detector/RotZ                   = 0 deg
d:Ge/Detector/HLX                    = 5 cm
d:Ge/Detector/HLY                    = 5 cm
d:Ge/Detector/HLZ                    = 2.5 cm
i:Ge/Detector/XBins = 1000
i:Ge/Detector/YBins = 1000
i:Ge/Detector/ZBins = 100

s:Sc/Dose/Quantity    = "DoseToMedium"
s:Sc/Dose/Component   = "Detector"
b:Sc/Dose/OutputToConsole = "False"
s:Sc/Dose/IfOutputFileAlreadyExists = "Overwrite"
s:Sc/Dose/OutputType = "binary"
b:Sc/Dose/PreCalculateStoppingPowerRatios = "True" #CHANGE

s:Sc/Dose/OutputFile = "output"
```
